# Associations between periconceptional lifestyle behaviours and adverse pregnancy outcomes

**DOI:** 10.1186/s12884-021-03935-x

**Published:** 2021-07-07

**Authors:** Veronique Y.F. Maas, Marjolein Poels, Marije Lamain-de Ruiter, Anneke Kwee, Mireille N. Bekker, Arie Franx, Maria P.H. Koster

**Affiliations:** 1 Department of Obstetrics and Gynaecology, Erasmus MC, University Medical Centre Rotterdam, Doctor Molewaterplein 40, 3015 GD Rotterdam, the Netherlands; 2 Research Agency Care2Research, Mattenbiesstraat 133, 1087GC Amsterdam, the Netherlands; 3 Research Agency Frontida Analytics, Prinses Irenelaan 95, 3554HD Utrecht, the Netherlands; 4 Department of Obstetrics and Gynaecology, Division Woman and Baby, University Medical Centre Utrecht, Lundlaan 6, 3584 EA Utrecht, the Netherlands

**Keywords:** Periconception period, Lifestyle behaviours, Pregnancy outcome, Risk factors, Preconception care

## Abstract

**Background:**

While the potential adverse outcomes of prenatal exposure to unhealthy lifestyle are widely evidenced, little is known about these exposures in the periconception period. We investigated the associations between lifestyle behaviours and adverse pregnancy outcomes with a unique distinction between preconceptional- and prenatal lifestyle behaviours.

**Methods:**

A secondary analysis took place within a prospective multicentre cohort study in the Netherlands, including 3,684 pregnant women. Baseline characteristics and preconceptional and first trimester lifestyle behaviours were assessed through a self-administered questionnaire in the first trimester. Adverse pregnancy outcomes (hypertensive disorders in pregnancy (HDP), small for gestational age (SGA), gestational diabetes (GDM) and spontaneous preterm birth (sPTB)) were reported by healthcare professionals. Data were collected between 2012 and 2014 and analysed using multivariate logistic regression.

**Results:**

Women who are overweight, and especially obese, have the highest odds of developing any adverse pregnancy outcome (adjusted odds ratio (aOR) 1.61 (95 % Confidence Interval (CI) 1.31–1.99) and aOR 2.85 (95 %CI 2.20–3.68), respectively), particularly HDP and GDM. Women who prenatally continued smoking attained higher odds for SGA (aOR 1.91 (95 %CI 1.05–1.15)) compared to the reference group, but these odds decreased when women prenatally quit smoking (aOR 1.14 (95 %CI 0.59–2.21)). Women who did not use folic acid supplements tended to have a higher odds of developing adverse pregnancy outcomes (aOR 1.28 (95 %CI 0.97–1.69)), while women who prenatally started folic acid supplements did not (aOR 1.01 (95 %CI 0.82–1.25)).

**Conclusions:**

Our results indicate that smoking cessation, having a normal body mass index (BMI) and initiating folic acid supplements preconceptionally may decrease the risk of adverse pregnancy outcomes. Therefore, intervening as early as the preconception period could benefit the health of future generations.

**Supplementary Information:**

The online version contains supplementary material available at 10.1186/s12884-021-03935-x.

## Background

Despite major advances in clinical research and medical technology, the prevalence of adverse maternal and neonatal pregnancy outcomes, such as preeclampsia and preterm birth, has only moderately decreased over the recent decades [1]. Adverse pregnancy outcomes are associated with long-term effects on health, for example, hypertensive disorders of pregnancy (HDP) and gestational diabetes (GDM) are independently associated with an increased risk for cardiovascular disease and type II diabetes [2]. Likewise, adverse neonatal outcomes, for example, preterm birth and small for gestations age (SGA), can have long term consequences among surviving infants, such as medical disabilities, impaired cognitive development, learning difficulties and behavioural- and psychological problems [3]. Evidence suggests that lifestyle changes, such as reducing alcohol use and smoking, losing weight, improving fruit and vegetable consumption, can reduce the prevalence of adverse pregnancy outcomes, especially when initiated in early pregnancy or even before conception [4–7].

The periconception period, defined as the 14 weeks before and 10 weeks after conception, is a critical window with a substantial impact on fetal growth and development [8]. Within this period, gametogenesis, organogenesis and placental development occur. These processes are vulnerable to disturbance of epigenetic mechanisms, leading to an altered profile of embryonic gene expression that persists throughout both pregnancy and childhood [9]. Tobacco- and alcohol consumption are two of the most critical teratogens for prenatal development [10, 11]. According to Dutch guidelines, the prevalence of women who continue smoking and alcohol consumption in at least the first trimester of pregnancy remains 7 % and approximately 40 %, respectively [12]. Moreover, in many Western countries, up to 50 % of women are overweight or obese when they become pregnant [13]. The maternal metabolic environment of women who are overweight or obese tends to affect placental development and these women are therefore more prone to develop adverse pregnancy outcomes like GDM or pre-eclampsia [14].

Health promotion activities, such as education, advice and a general health assessment, are likely to improve pregnancy outcomes, by early identification of risk factors encouraging behavioural change [15–17]. One way to incorporate health promotion activities in maternity care is through preconception care (PCC). Several studies aimed to implement PCC programs and some successfully led to an improved level of knowledge regarding PCC and subsequent improved periconceptional lifestyle behaviours [7, 18–20]. The potential effect of healthy periconceptional lifestyle behaviours on reducing multiple adverse pregnancy outcomes together is yet understudied, and it has been studied only in small sample sizes [7, 21]. Therefore, the objective of our study was to investigate the association between periconceptional lifestyle behaviours and adverse pregnancy outcomes with a unique distinction between preconceptional- and prenatal lifestyle behaviours.

## Methods

### Design

From December 2012 through January 2014, a prospective multicentre cohort study was conducted in the central region of the Netherlands; the RESPECT (Risk EStimation for PrEgnancy Complications to provide Tailored care) study. The initial aim of the RESPECT study was to perform an external validation and direct comparison of published prognostic models for early prediction of the risk of developing adverse pregnancy outcomes, including predictors applicable in the first trimester of pregnancy [22]. The current analysis is a secondary analysis of the RESPECT study.

### Setting

Participants were recruited in 31 independent midwifery practices (primary care) and six hospitals (secondary/tertiary care). All pregnant women less than 14 weeks of gestation were included at their initial prenatal visit; there were no exclusion criteria for participating in the RESPECT study. A detailed description of the cohort has previously been published [22, 23]. The RESPECT study has been approved by the Medical Ethics Committee of the University Medical Centre Utrecht (protocol no. 12–432/C) and written informed consent was obtained from all individual participants.

### Sample

**Fig. 1:**
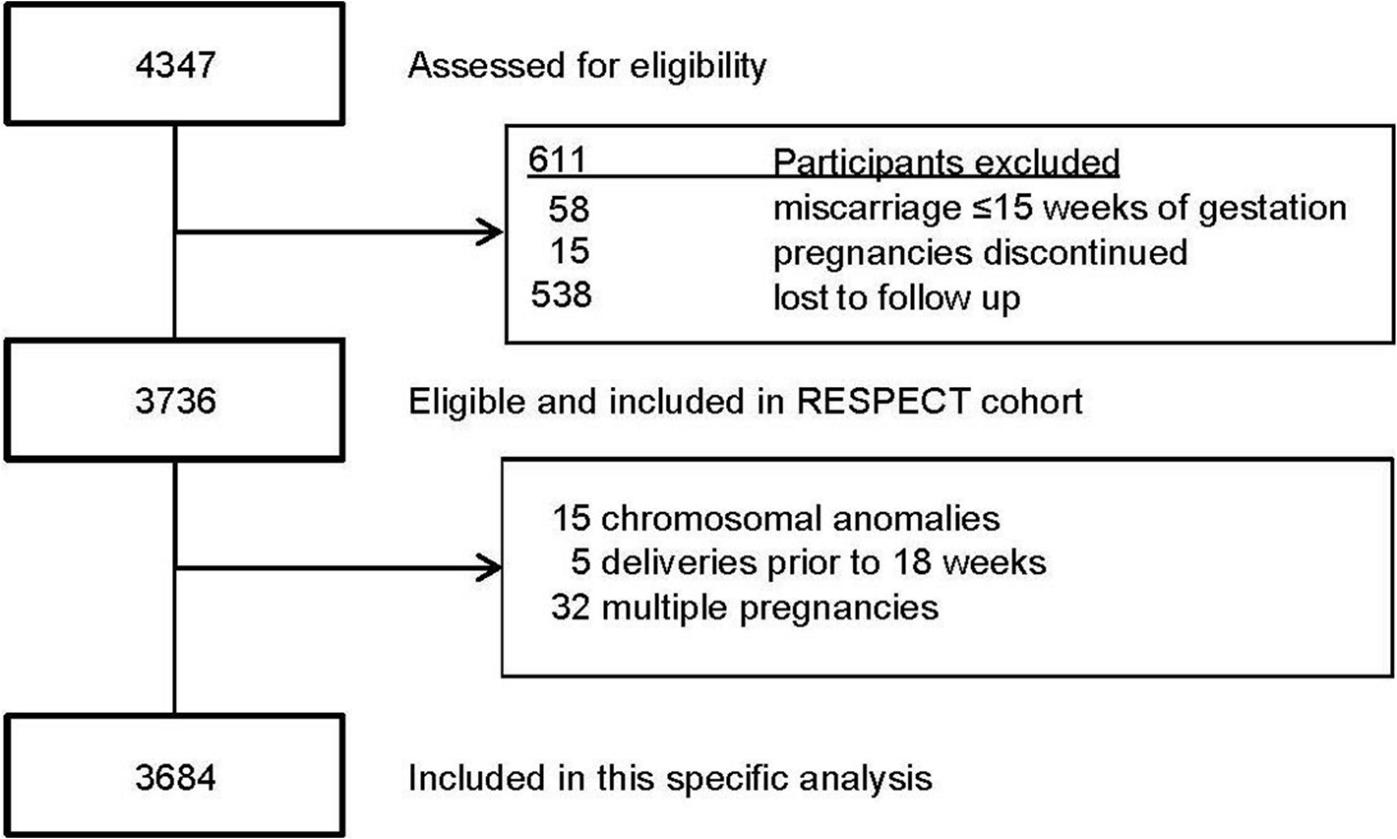
Flowchart of study participants

In total, 4,347 pregnant women participants from the RESPECT study were assessed for eligibility, women with miscarriages before 15 weeks of gestation, women who discontinued their pregnancy or were lost to follow-up were not eligible. For this specific analysis, we excluded women with pregnancies complicated by chromosomal anomalies, births before 18 weeks, multiple pregnancies after multiple imputation (see statistical paragraph). Hence, 3,684 participants were included in this specific analysis as visible in Fig. 1.

### Measures

At enrolment in the first trimester of pregnancy, women were asked to fill out a questionnaire specifically designed for this study. This self-reported questionnaire contained items on socio-demographic characteristics, lifestyle behaviours, and medical, family and obstetrical history. In the questionnaire, women were asked to recall their lifestyle behaviours in the three-month period before conception and then again for their current lifestyle behaviours (in the first trimester). We were therefore able to measure a difference in lifestyle behaviours for the following lifestyle behaviours: use of tobacco and alcohol and the use of supplements (folic acid, vitamin C, vitamin D, calcium or multivitamin). After birth, healthcare professionals reported the presence or absence of pregnancy outcomes through standardized forms. The definitions of lifestyle behaviours, sociodemographic characteristics and adverse pregnancy outcomes are shown in Table 1. The use of any vitamin or calcium supplement were combined and analysed as one determinant called ‘vitamin use’. As multivitamin includes folic acid, women using multivitamin were categorized in both ‘folic acid use’ and ‘vitamin use’. Body mass index (BMI) was calculated based on self-reported answers to questions concerning one’s height and weight before conception. Even though BMI itself is not a lifestyle behaviour, certain lifestyle behaviours such as diet or exercise can influence a person’s BMI. The following socio-demographic characteristics were assessed: age, ethnicity, educational level, parity and mode of conception. The following pregnancy outcomes were assessed: hypertensive disorder in pregnancy (HDP; either pregnancy-induced hypertension or preeclampsia), small for gestational age (SGA) defined as birth weight < 3rd percentile, gestational diabetes (GDM), and spontaneous preterm birth (sPTB). Table 1 provides details on how these pregnancy outcomes were defined. The choice for these specific pregnancy- and neonatal outcomes was based on its prevalence, the relevance for both mother and child and its need for preventive intervention early in pregnancy. Participants were classified into either having experienced an uncomplicated pregnancy or being diagnosed with any adverse pregnancy outcome, included in our composite outcome. In case of more than one adverse pregnancy outcome, women were assigned to multiple groups. However, since SGA is a common consequence of HDP and these adverse outcomes are therefore likely to co-exist with each other, women with both these outcomes were only assigned to the HDP group. Women were assigned to the SGA group when SGA was the only adverse outcome.

**Table 1:** Overview of demographics and pregnancy outcomes with their definitions

	Variable	Definition
**Lifestyle Behaviours** (Self-reported in the first trimester)	Body mass index	Kg/m^2^
	Underweight	<18.5 kg/m^2^
	Normal weight	18.5 - 25 kg/m^2^
	Overweight	25-30 kg/m^2^
	Obese	>30 kg/m^2^
	Daily fruit intake	Pieces of fruit
	Not-adequate	< 2 pieces
	Adequate	≥ 2 pieces
	Tobacco use	≥1 cigarette per day (yes/no)
	Alcohol use	≥1 glass of alcohol per week (yes/no)
	Folic acid use	Use of folic acid, either as a single supplement or as part of multivitamin preparation (yes/no)
	Vitamin use	Use of vitamin C, vitamin D or calcium supplement either as a single supplement or as part of multivitamin preparation (yes/no)
**Socio-demographic characteristics** (Self-reported in the first trimester)	Age	Years
	Ethnicity	- White: Caucasian or other Western;
		- Non-White: African, Hindustani, Moroccan, Turkish, Middle Eastern, Asian, other non-western, and mixed.
	Educational level	- Low: primary education or lower level;
		- Medium: secondary education;
		- High: tertiary education or higher level.
	Parity	- Nulliparous: women with no previous pregnancies beyond 16 weeks;
		- Multiparous: women with previous pregnancies beyond 16 weeks.
	Mode of conception	- Spontaneous conception: pregnant without medical assistance;
		- Non-spontaneous conception: pregnant with ovulation drugs, insemination or in vitro fertilization/intracytoplasmic sperm injection (IVF/ICSI).
**Adverse pregnancy outcomes** (Reported by the healthcare provider after the pregnancy)	Hypertensive disorder in pregnancy (HDP)	Either:
		- Pregnancy-induced hypertension (PIH): the new onset of hypertension (≥ 140 mmHg systolic and/or ≥90 mmHg diastolic blood pressure) after 20 weeks gestation measured on at least two occasions four hours apart;
		- Pre-eclampsia (PE): PIH accompanied by proteinuria (≥300 mg in 24 hours), either during pregnancy or postpartum [24, 25, 26].
	Small for gestation age (SGA)	A birth weight <3rd percentile, based on Hoftiezer percentiles [27, 28].
	Gestational diabetes (GDM)	The presence of either a fasting glucose level of ≥7.0 mmol/L (126 mg/dl) or a glucose level of ≥7.8 mmol/L (140 mg/dl) two hours after a 75-grams oral glucose tolerance test [29–31].
	Spontaneous preterm birth (sPTB)	A delivery with spontaneous onset before 37 weeks of gestation [32].
	Composite outcome	Women with one of the following complications; pregnancy-induced hypertension, pre-eclampsia, small for gestational age <p3, gestational diabetes, spontaneous preterm birth or fetal death.

### Statistical analysis

The original dataset contained missing data for some participants; there were 1,111 cases (30.2 %) with at least one missing value. A more detailed description and an assessment of these missing values can be found in the Appendix (Additional File 1). Missing values were imputed using multiple imputation [22, 33]. All variables and outcomes were used in the imputation model and ten imputations were performed. The results shown are the results after multiple imputation. Rubin’s rules were applied to combine the results into summary estimates [34]. Baseline data for all participants are presented as medians and interquartile range (IQR) for continuous variables or as numbers and percentages for categorical variables. Logistic regression analysis was performed to identify associations between lifestyle behaviours and adverse pregnancy outcomes. Crude odds ratios (OR) and accompanying 95 % confidence intervals (CI) were calculated by univariate analysis. Subsequently, adjusted ORs were calculated by multivariate analysis, taking potential confounders into account (maternal age, educational level, ethnicity, parity and mode of conception). Reference categories were chosen for categorical variables based on the desired lifestyle behaviour based on the Dutch guidelines and the recommended daily intake of vitamins [12, 31]. The statistical analysis of the data was performed in the final months of 2019, using SPSS version 25.0. *P*-values < 0.05 were considered statistically significant.

## Results

The median age of the participants in our cohort was 30.8 years (IQR 28.0-33.6) and 3,330 (90.4 %) women were Caucasian (Table 2). Conception occurred spontaneously in 3,429 (93.1 %) women, 2,131 (57.8 %) women were highly educated and 1,643 (44.6 %) women were nulliparous. The proportion of women who smoked and used alcohol preconceptionally was 20.9 % (*n* = 771) and 60.9 % (*n* = 2,244), respectively. The majority of these women changed their unhealthy lifestyle behaviours in the first trimester, 492 women (63.8 %) quit smoking and 2,216 women (98.7 %) quit drinking alcohol. A total of 2,177 (59.1 %) women started using folic acid supplements preconceptionally, while 1,077 (29.2 %) women started using folic acid supplement after conception took place.

**Table 2:** Demographics and lifestyle behaviours of study participants stratified by pregnancy outcome

	Cohort	Uncomplicated	Adverse pregnancy outcome				
			Composite	HDP	GDM	sPTB	SGA
	*n*=3684	*n*=2972 (80.7)	*n*=712 (19.3)	*n* = 298 (8.1)	*n*=184 (5.0)	*n*=127 (3.4)	*n*=133 (3.6)
Age (years)	30.8 (28.0-33.6)	30.8 (27.9-33.6)	30.8 (28.2 -33.7)	30.3 (27.6-32.9)	31.8 (29.7-34.8)*	30.6 (28.2-32.6)	30.6 (28.0-33.9)
Ethnicity							
Caucasian	3330 (90.4)	2706 (91.0)	624 (87.6)	277 (93.0)	144 (78.3)	117 (92.1)	111 (83.5)
Non-Caucasian	354 (9.6)	266 (9.0)	88 (12.4)*	21 (7.0)	40 (21.7)	10 (7.9)	22 (16.5)*
Educational level							
Low	279 (7.6)	213 (7.2)	66 (9.3)*	20 (6.7)	18 (9.8)	11 (8.7)	20 (15.0)*
Medium	1274 (34.6)	995 (33.5)	279 (39.3)*	124 (41.6)*	82 (44.6)*	47 (37.0)	49 (36.8)
High	2131 (57.8)	1764 (59.3)	367 (51.4)	154 (51.7)	84 (45.7)	69 (54.3)	64 (48.2)
Parity							
Nullipara	1643 (44.6)	1224 (41.2)	418 (58.8)*	189 (63.4)*	73 (39.7)	86 (67.7)*	34 (25.6)*
Multipara	2041 (55.4)	1748 (58.8)	293 (41.2)	109 (36.6)	110 (59.8)	41 (32.3)	99 (74.4)
Mode of conception							
Spontaneous conception	3429 (93.1)	2791 (93.9)	638 (89.6)	261 (87.6)	155 (84.2)	116 (91.4)	120 (90.2)
Non- spontaneous conception	255 (6.9)	181 (6.1)	74 (10.4)*	37 (12.4)*	29 (15.8)*	11 (8.7)	13 (9.8)
Pre-pregnancy BMI							
Underweight (<18.5 kg/m^2^)	99 (2.7)	81 (2.7)	18 (2.5)*	2 (0.7)	2 (1.1)	4 (3.1)	9 (6.8)*
Normal weight (18.5-25 kg/m^2^)	2367 (64.3)	2001 (67.4)	367 (51.6)	137 (45.9)	67 (36.4)	86 (67.7)	86 (64.6)
Overweight (25-30 kg/m^2^)	852 (23.1)	654 (22.0)	198 (27.8)*	97 (32.6)*	57 (31.0)*	28 (22.0)	25 (18.8)
Obese (>30 kg/m^2^)	366 (9.9)	236 (7.9)	129 (18.1)*	62 (20.8)*	57 (31.0)*	9 (7.1)	13 (9.8)
Smoking							
No preconception smoking	2913 (79.0)	2388 (80.3)	526 (73.8)	224 (75.2)	140 (76.1)	86 (67.7)	92 (69.2)
Smoked, quit prenatal	492 (13.4)	371 (12.5)	120 (16.9)*	53 (17.8)	29 (15.8)	26 (20.5)*	20 (15.0)*
Smoked, did not quit prenatal	279 (7.6)	213 (7.2)	66 (9.3)	21 (7.0)	15 (8.2)	15 (11.8)*	21 (15.8)
Alcohol use							
No preconception use	1440 (39.1)	1138 (38.3)	302 (42.4)	119 (39.9)	101 (54.9)	48 (37.8)	55 (41.4)
Used alcohol, quit prenatal	2216 (60.1)	1810 (60.9)	406 (57.0)	178 (59.7)	83 (45.1)*	79 (62.2)	76 (57.1)
Used alcohol, did not quit prenatal	28 (0.8)	24 (0.8)	4 (0.6)	1 (0.4)	0 (0)	0 (0)	2 (1.5)
Folic acid use^b^ (incl. multivitamin)							
No preconception use	430 (11.7)	330 (11.1)	99 (13.9)	42 (14.1)	27 (14.7)	15 (11.8)	20 (15.0)
Only prenatal	1077 (29.2)	879 (29.6)	198 (27.8)	77 (25.8)	50 (27.2)	37 (29.1)	42 (31.6)
Preconception and prenatal	2177 (59.1)	1763 (59.3)	415 (58.3)	179 (60.1)	107 (58.2)	75 (59.1)	71 (53.4)
Vitamin use^b^							
No preconception use	1276 (34.6)	998 (33.6)	279 (39.2)	123 (41.3)	65 (35.3)	44 (34.6)	61 (45.8)
Only prenatal	1405 (38.1)	1161 (39.0)	243 (34.1)	96 (32.2)	68 (37.0)	44 (34.6)	40 (30.1)
Preconception and prenatal	1003 (27.3)	813 (27.4)	190 (26.7)	79 (26.5)	51 (17.7)	39 (30.7)	32 (24.1)
Fruit							
Daily fruit intake < 2 pieces	1409 (38.2)	1124 (37.8)	285 (40.0)	122 (40.9)	71 (38.6)	46 (36.2)	56 (42.1)
Daily fruit intake ≥ 2 pieces	2275 (61.8)	1849 (62.2)	427 (60.0)	176 (59.1)	112 (60.9)	81 (63.8)	77 (57.9)

values are presented as median (IQR) or n (%)

participants can have more than 1 adverse pregnancy outcome therefore the numbers do not add up

*HDP* hypertensive disorders in pregnancy, *GDM* gestational diabetes, *sPTB* spontaneous preterm birth, *SGA* small for gestational age

*differs significantly from the uncomplicated group based on logistic regression analysis, *p*<0.05

^a^composite outcome: women with one of the following complications; HDP, SGA, GDM, sPTB or fetal death

^b^women using multivitamin were categorized in both ‘folic acid use’ and ‘vitamin use’

Table 2 also shows the demographic characteristics and lifestyle behaviours of women who experienced an uncomplicated pregnancy (*n* = 2,972; 80.7 %) versus women who experienced an adverse pregnancy outcome: HDP (*n* = 298; 8.1 %), SGA (*n* = 133; 3.6 %), GDM (*n* = 184; 5.0 %), sPTB (*n* = 127; 3.4 %). In total 712 (19.3 %) of all women experienced an adverse pregnancy outcome. These adverse pregnancy outcomes appeared significantly more often when women were non-Caucasian, were low- or medium educated, were nulliparous, had a non-spontaneous conception, were either under-or overweight or smoked preconceptionally.

Table 3 shows the associations between lifestyle behaviours and all adverse pregnancy outcomes. Overall, women who are overweight or obese and women who smoked preconceptionally had higher odds of developing adverse pregnancy outcomes with an adjusted OR of 1.61 (95 %CI 1.13–1.99), 2.85 (95 %CI 2.20–3.68) and 1.32 (95 %CI 1.03–1.71), respectively. Women with obesity had the highest odds of developing GDM (aOR 6.85 (95 %CI 4.39–10.71)), these increased odds remain in women who are overweight, although much lower (aOR 2.38 (95 %CI 1.60–3.54)). Women who are overweight or obese were also more likely to develop HDP (aOR 2.17 (95 %CI 1.63–2.89) and 3.80 (95 %CI 2.68–5.40), respectively). In contrast, women with a pre-pregnancy BMI of ≤ 18.5 kg/m^2^ had a higher odds of SGA (aOR 2.64 (95 %CI 1.17–5.96). We found that women who smoked before pregnancy were more likely to experience sPTB (aOR 1.76 (95 %CI 1.05–2.93)) compared to women who did not smoke preconceptionally. Women who continued to smoke during pregnancy were also more likely to give birth to an SGA neonate aOR 1.91 (95 %CI 1.05–1.15), which was not the case for women who quit smoking after conception (aOR 1.14 (95 %CI 0.59–2.21)). Women who consumed alcohol preconceptionally, yet discontinued in the first trimester, had a lower odds of developing GDM compared to women who were not used to drink alcohol before pregnancy recognition (aOR 0.65 (95 % CI 0.46–0.93)). Compared to women who used folic acid supplements from the preconception period onwards, women who did not use folic acid supplements tended to have a (non-significantly) higher odds of developing adverse pregnancy outcomes (aOR 1.28 (95 %CI 0.97–1.69)), while women who started folic acid supplements during pregnancy did not (aOR 1.01 (95 %CI 0.82–1.25)). No associations were found between daily fruit intake or vitamin use and the development of adverse pregnancy outcomes.

**Table 3:** Associations between lifestyle behaviours and adverse pregnancy outcome

	Composite (***n***=712)^**a**^		HDP (***n***=298)		GDM (***n***=184)		sPTB (***n***=127)		SGA (***n***=133)	
	Crude OR (95% CI)	Adjusted OR (95% CI)^b^	Crude (95% CI)	Adjusted (95% CI)^b^	Crude (95% CI)	Adjusted (95% CI)^b^	Crude (95% CI)	Adjusted (95% CI)^b^	Crude (95% CI)	Adjusted (95% CI)^b^
Pre-pregnancy BMI										
Underweight (<18.5 kg/m^2^)	1.18 (0.66-2.12)	1.22 (0.67 - 2.23)	0.43 (0.11-1.74)	0.44 (0.11-1.82)	0.86 (0.20-3.68)	0.92 (0.21-4.04)	1.07 (0.32-3.60)	1.10 (0.32-3.79)	2.65 (1.23-5.74)	2.64 (1.17-5.96)
Normal weight (18.5-25 kg/m^2^)	ref	ref	ref	ref	ref	ref	ref	ref	ref	ref
Overweight (25-30 kg/m^2^)	1.65 (1.35-2.03)	1.61 (1.31 - 1.99)	2.18 (1.5-2.89)	2.17 (1.63-2.89)	2.59 (1.77-3.80)	2.38 (1.60-3.54)	1.01 (0.63-1.61)	0.99 (0.61-1.60)	0.89 (0.52-1.51)	0.84 (0.48-1.46)
Obese (>30 kg/m^2^)	2.96 (2.31-3.80)	2.85 (2.20 - 3.68)	3.83 (2.72-5.38)	3.80 (2.68-5.40)	7.18 (4.74 - 10.89)	6.85 (4.39-10.71)	0.85 (0.37-1.93)	0.82 (0.36 - 1.85)	1.29 (0.67-2.50)	1.16 (0.59 - 2.31)
Smoking										
no preconception use	ref	ref	ref	ref	ref	ref	ref	ref	ref	ref
smoked, quit prenatal	1.48 (1.16-1.87)	1.32 (1.03 - 1.70)	1.52 (1.08-2.14)	1.33 (0.94-1.90)	1.33 (0.87-2.05)	1.24 (0.79-1.95)	1.93 (1.19-3.13)	1.76 (1.05-2.93)	1.38 (0.75-2.53)	1.14 (0.59-2.21)
smoked, did not quit prenatal	1.41 (1.03-1.94)	1.23 (0.86-1.75)	1.03 (0.61-1.72)	0.88 (0.52-1.50)	1.17 (0.64-2.13)	1.05 (0.55-2.02)	1.91 (1.04-3.49)	1.83 (0.91-3.65)	2.47 (1.45-4.21)	1.91 (1.05 - 1.15)
Alcohol consumption										
no preconception use	ref	ref	ref	ref	ref	ref	ref	ref	ref	ref
used alcohol, quit prenatal	0.85 (0.70-1.02)	0.84 (0.69 - 1.03)	0.94 (0.72-1.24)	0.87 (0.65-1.15)	0.51 (0.37-0.71)	0.65 (0.46-0.93)	1.03 (0.71-1.51)	0.89 (0.60-1.34)	0.88 (0.60-1.31)	0.88 (0.57-1.36)
used alcohol, did not quit prenatal^c^	0.58 (0.17-1.92)	0.57 (0.17-1.94)	_	_	_	_	_	_	_	_
Folic acid use^de^										
no preconception use	1.28 (0.98-1.65)	1.28 (0.97-1.69)	1.26 (0.87-1.81)	1.48 (1.00-2.18)	1.33 (0.84-2.11)	0.97 (0.58-1.62)	1.02 (0.54-1.92)	1.18 (0.59-2.35)	1.51 (0.86-2.63)	1.41 (0.75-2.64)
only prenatal	0.96 (0.79-1.17)	1.01 (0.82-1.25)	0.85 (0.63-1.14)	0.95 (0.70-1.31)	0.93 (0.65-1.33)	0.96 (0.66-1.40)	0.99 (0.65-1.50)	1.10 (0.70-1.71)	1.17 (0.77-1.76)	1.26 (0.81-1.96)
preconception and prenatal	ref	ref	ref	ref	ref	ref	ref	ref	ref	ref
Vitamin use^ef^										
no preconception use	1.19 (0.96-1.48)	1.19 (0.95-1.50)	1.26 (0.93-1.72)	1.36 (0.99-1.87)	1.05 (0.71-1.55)	0.91 (0.60-1.38)	0.92 (0.58-1.45)	0.95 (0.59-1.54)	1.53 (0.94-2.47)	1.48 (0.88-2.49)
only prenatal	0.90 (0.72-1.11)	0.89 (0.72-1.12)	0.85 (0.61-1.17)	0.86 (0.61-1.20)	0.95 (0.64-1.39)	0.99 (0.67-1.46)	0.78 (0.50-1.22)	0.78 (0.49-1.22)	0.87 (0.53-1.44)	0.87 (0.53-1.46)
preconception and prenatal	ref	ref	ref	ref	ref	ref	ref	ref	ref	ref
Fruit										
daily fruit intake < 2 pieces	0.91 (0.76-1.08)	0.84 (0.70-1.01)	0.87 (0.73-1.14)	0.79 (0.60-1.04)	0.96 (0.69-1.32)	0.92 (0.66-1.29)	1.08 (0.74-1.59)	0.96 (0.64-1.44)	0.84 (0.58-1.21)	0.72 (0.49-1.06)
daily fruit intake ≥ 2 pieces	ref	ref	ref	ref	ref	ref	ref	ref	ref	ref

*HDP* hypertensive disorders in pregnancy, *GDM* gestational diabetes, *sPTB* spontaneous preterm birth, *SGA* small for gestational age, *OR* odds ratio, *CI* confidence interval

^a^composite outcome: women with one of the following complications; HDP, GDM, sPTB, SGA or fetal death.

^b^adjusted for non-modifiable factors (age, ethnicity, educational level, nulliparity, spontaneous conception)

^c^n was too small in total population (*n*=28) to calculate odds ratios

^d^including multivitamin use

^e^women using multivitamin were categorized in both ‘folic acid use’ and ‘vitamin use’

^f^vitamin C, vitamin D, calcium or multivitamin use

## Discussion

This study confirms that unhealthy periconceptional lifestyle behaviours are associated with a higher prevalence of adverse pregnancy outcomes showing a unique distinction between preconceptional- and prenatal lifestyle behaviours. Women who were obese before pregnancy had the highest odds of developing adverse pregnancy outcomes, particularly HDP and GDM. These odds were much lower in women who are overweight. Women who are underweight, on the other hand, were more likely to give birth to an SGA neonate. Smoking before the pregnancy was associated with sPTB and SGA, but, interestingly, for SGA this association did not persist when women quit smoking during the first trimester. Therefore, encouraging women to quit smoking in the first trimester could reduce the odds for SGA to those of a non-smoker.

We found that women with a BMI of 25 kg/m^2^ or more, especially women with a BMI above 30 kg/m^2^, have the highest odds of developing adverse pregnancy outcomes. Improving one’s exercising pattern through health-promoting interventions could benefit these women as previous meta-analyses suggest that higher amounts of preconception physical activity are associated with a lower risk of GDM and pre-eclampsia [35, 36]. In addition, a population-based study showed that a 10 % lower preconception BMI was associated with clinically meaningful risk reduction in pre-eclampsia, GDM, preterm birth, macrosomia, and stillbirth [4]. A previous study showed that only 57 % of pregnant women were aware of the fact that obesity increases the overall risk of pregnancy- and birth complications and that weight loss before the pregnancy can reduce to overall risk for complications [37]. Hence, here lies an opportunity for PCC to encourage women with obesity to enter weight loss programs to improve their own health and the health of their future child.

In accordance with previous studies, we indeed found that smoking is associated with a higher odds of sPTB and birth of SGA neonates [38–41]. Although some studies showed that pregnant women who quit smoking during the first trimester are no longer at risk for sPTB, our results show that women who quit smoking and women who continue to smoke (although not significant) both have increased odds for sPTB [42–44]. On the other hand, we found that women who quit smoking in the first trimester did have similar odds of developing SGA compared to non-smokers, as previous studies have also suggested [40, 41, 43]. Cigarette smoke contains substances that affect placental endothelial function, which can lead to the development of ischemic vascular changes impacting placental growth and functions [45]. A previous systematic review showed that cessation of smoking before and shortly after becoming pregnant was not associated with SGA and this suggests that the mechanisms affecting fetal growth predominantly act beyond the first trimester [46]. Nevertheless, smoking cessation before conception remains the best approach to improve health benefits.

Alcohol is suggested to lower levels of inflammation markers and endothelial dysfunction, increase insulin sensitivity, increase HDL cholesterol concentrations, which, for example, may lower the risk of type 2 diabetes mellitus and possibly also GDM [47–49]. Several previous studies indicated that (heavy) alcohol consumption during pregnancy increases the risk of low birth weight and preterm birth, while light alcohol consumption may possibly have a mildly protective effect on these outcomes [50, 51]. The possible explanation provided for this paradox is the “healthy drinker effect”, in which women with poor obstetric prognosis, socio-economic status or well-being are more likely to abstain from drinking alcohol [50, 51]. While we were not able to measure the amount of alcohol consumed, we indeed found that women who used alcohol preconceptionally were significantly more often Caucasian, higher educated, nulliparous, were pregnant by spontaneous conception, had a lower pre-pregnancy BMI and used more folic acid supplements compared to women who did not use alcohol preconceptionally (data not shown).

Finally, our results showed that encouraging women to start folic acid supplements, after pregnancy recognition, can still benefit the health of mother and child. Although non-significant, we found a higher odds of adverse pregnancy outcomes for women who did not use folic acid supplements compared to preconceptional commencement. Although we found no difference in adverse pregnancy outcomes between women who started folic acid supplements before or during the pregnancy, it is widely evidenced that early initiation (ideally before conception) of folic acid supplements does decrease the risk for congenital malformations such as neural tube defects [52]. In our study, congenital malformations were excluded from analysis and therefore we cannot provide any results regarding these outcomes.

Our results are relevant since an unhealthy diet, lifestyle behaviours and exercising pattern are progressively becoming part of Western society, including among a high percentage of women in their reproductive age [5, 6, 16]. Encouraging women to develop and maintain a healthy lifestyle has long been a focus of prenatal care, while our findings support emerging evidence indicating that the preconception period might even be a better window of opportunity to address these unhealthy lifestyle behaviours. PCC is known to increase the health and well-being of prospective parents, still, the uptake of PCC-consults remains remarkably low [53]. This is particularly the case for vulnerable women, who often have multiple unhealthy lifestyle behaviours and are specifically hard to reach [54]. PCC interventions often require engagement from prospective parents who are not yet thinking about becoming a parent in the future and are not yet known by maternal health services [55]. Although some studies suggest that awareness of preconception health and care is low, pregnancy planning appears relatively common, indicating a missed and unexploited opportunity for intervention [56–58].

A possible strength of this study is that we used a large, multicentre, population-based cohort where we accounted for missing data by using multiple imputation, which decreases the risk of selection bias and allows to investigate multiple exposures and outcomes. Also, we distinguished lifestyle behaviours between the preconception period and the first trimester, a distinction rarely made in previous studies. A limitation of this study is the underrepresentation of non-caucasian and lower educated women. Future studies should focus on analysing these associations in a more heterogeneous study population. In addition, the data on lifestyle behaviours is collected by the use of self-administered questionnaires. Although this method is suggested to negatively affect the validity, we merely assessed the presence (yes/no questions) instead of frequencies of lifestyle behaviours, by which we probably have diminished the chance of over-or underreporting of behaviour [59]. However, to be able to make the distinction between preconception and first trimester exposure to unhealthy lifestyle behaviours depends upon women’s perception of when conception exactly occurred. Moreover, examining potential dose-response relationships was not possible and blood markers were not available to validate, for example, micronutrient or smoking status. Finally, as this study was only able to measure associations between periconceptional lifestyle behaviours and adverse pregnancy outcomes, and the sample size calculation was not performed for the current aim of this paper, results should be interpreted with caution and we recommend future research to focus on large-scale interventions to discover a possible (causal) effect.

## Conclusions

Overall, our findings indicate that women should be encouraged to change unhealthy lifestyle behaviours, preferably before conception. Therefore, future research on interventions to improve awareness of the importance of PCC and the (cost)effectiveness of these interventions on pregnancy outcomes are needed. Findings from such studies could enhance the choice to start future preventive measures and interventions regarding unhealthy periconceptional lifestyle behaviours, to optimize the health of future generations.

## Supplementary Information

**Additional file 1.** Baseline characteristics stratified by variables that were available for imputation

## Data Availability

The dataset for the current study is available from the corresponding author upon reasonable request.

## Abbreviations

aOR: Adjusted odds ratio
BMI: Body mass index
CI: Confidence interval
GDM: Gestational diabetes
HDP: Hypertensive disorders in pregnancy
IQR: Interquartile range
PCC: Preconception care
PE: Pre-eclampsia
PIH: Pregnancy-induced hypertension
RESPECT study: Risk EStimation for PrEgnancy Complications to provide Tailored care
SGA: Small for gestational age
sPTB: Spontaneous preterm birth
