# Supplementary material for: Associations between periconceptional lifestyle behaviours and adverse pregnancy outcomes
Source: BMC Pregnancy Childbirth. 2021 Jul 7;21:492. doi: 10.1186/s12884-021-03935-x (PMC8265143; doi:10.1186/s12884-021-03935-x)
Supplement: Supplementary file 1 — Additional file 1. Baseline characteristics stratified by variables that were available for imputation [file 12884_2021_3935_MOESM1_ESM.docx]

**Associations between** **periconceptional lifestyle behaviours and adverse pregnancy outcomes**

Veronique Y.F. Maas^a^, Marjolein Poels^a,b^, Marije Lamain-de Ruiter^c^, Anneke Kwee^d^, Mireille N. Bekker^d^, Arie Franx^a^, Maria P.H. Koster^a^.

^a^ Erasmus MC, University Medical Centre Rotterdam, Department of Obstetrics and Gynaecology, Doctor Molewaterplein 40, 3015 GD Rotterdam, the Netherlands.

^b^ Research agency Care2Research, Mattenbiesstraat 133, 1087GC, Amsterdam, the Netherlands.

^c^ Research agency Frontida Analytics, Prinses Irenelaan 95, 3554HD, Utrecht, the Netherlands.

^d^ University Medical Centre Utrecht, department of Obstetrics and Gynaecology, Division Woman and Baby, Lundlaan 6, 3584 EA Utrecht, the Netherlands

**Correspondence:**

dr. M.P.H. (Wendy) Koster

Department of Obstetrics and Gynaecology

Erasmus MC, University Medical Centre Rotterdam

Doctor Molewaterplein 40, 3015 GD Rotterdam, The Netherlands

E-mail: [m.p.h.koster@erasmusmc.nl](mailto:m.p.h.koster@erasmusmc.nl)

**Additional file 1. Baseline characteristics stratified by variables that were available for imputation**

| **Characteristic** | **Missing^1^ n (%)** | **Complete questionnaires^1^ (n=2576)** | **Cases with ≥ 1 missing value^1^** | **Overall RESPECT cohort^2^**  **(n=3684)** |
| --- | --- | --- | --- | --- |
|  |  |  | **(n=1111)** |  |
| *Demographic characteristics* |  |  |  |  |
| Age (median (IQR)) (in years) | 165 (4.5) | 30.8 (27.9-33.8) | 30.8 (28.1-33.2) | 30.8 (28.0-33.6) |
| Age (in years) | 165 (4.5) |  |  |  |
| <20 |  | 11 ( 0.5) | 5 ( 0.5) | 17 ( 0.5) |
| 20-25 |  | 218 ( 9.0) | 69 ( 6.2) | 300 ( 8.1) |
| 25-30 |  | 790 (32.8) | 392 (35.3) | 1238 (33.6) |
| 30-35 |  | 991 (41.1) | 498 (44.8) | 1553 (42.1) |
| ≥35 |  | 401 (16.6) | 147 (13.2) | 576 (15.6) |
| Ethnicity | 721 (19.6) |  |  |  |
| Caucasian |  | 1645 (88.7) | 1055 (95.0) | 3318 (90.1) |
| African |  | 18 ( 1.0) | 2 ( 0.2) | 32 ( 0.9) |
| Hindustani |  | 8 ( 0.4) | 0 ( 0.0) | 14 ( 0.4) |
| Moroccan |  | 49 ( 2.6) | 9 ( 0.8) | 74 ( 2.0) |
| Turkish |  | 45 ( 2.4) | 10 ( 0.9) | 72 ( 1.9) |
| Middle Eastern |  | 7 ( 0.4) | 4 ( 0.4) | 14 ( 0.4) |
| Asian |  | 30 ( 1.6) | 11 ( 1.0) | 56 ( 1.5) |
| Western |  | 5 ( 0.3) | 2 ( 0.2) | 12 ( 0.3) |
| Non-western |  | 4 ( 0.2) | 3 ( 0.3) | 10 ( 0.3) |
| Mixed |  | 44 ( 2.4) | 15 ( 1.4) | 83 ( 2.3) |
| Education | 218 (5.9) |  |  |  |
| High |  | 1343 (57.0) | 699 (62.9) | 2131 (57.9) |
| Moderate |  | 817 (31.7) | 360 (32.4) | 1274 (34.6) |
| Low |  | 198 ( 7.7) | 52 ( 4.7) | 279 ( 7.6) |
| *Health characteristics* |  |  |  |  |
| Prepregnancy BMI (median (IQR)) | 46 (1.3) | 23.3 (21.2-26.3) | 23.0 (20.9-25.9) | 23.2 (21.1-26.2) |
| Prepregnancy BMI | 46 (1.3) |  |  |  |
| Underweight (<18.5 kg/m^2^) |  | 70 ( 2.8) | 27 ( 2.4) | 99 ( 2.7) |
| Normal weight (18.5-25 kg/m^2^) |  | 1605 (63.4) | 739 (66.5) | 2367 (64.3) |
| Overweight (25-30 kg/m^2^) |  | 595 (23.5) | 245 (22.1) | 852 (23.1) |
| Obese (≥30 kg/m^2^) |  | 260 (10.3) | 100 ( 9.0) | 366 ( 9.9) |
| Chronic illness | 2 (0.05) | 97 ( 3.8) | 29 ( 2.6) | 126 ( 3.4) |
| high blood pressure | 1 (0.03) | 45 ( 1.7) | 15 ( 1.4) | 60 ( 1.6) |
| diabetes mellitus I/II | 1 (0.03) | 11 ( 0.4) | 2 ( 0.2) | 13 ( 0.4) |
| cardiovascular disease | 2 (0.05) | 20 ( 0.8) | 7 ( 0.6) | 27 ( 0.7) |
| thrombosis | 1 (0.03) | 9 ( 0.3) | 5 ( 0.5) | 14 ( 0.4) |
| systemic lupus erythematosus | 1 (0.03) | 1 ( 0.0) | 0 ( 0.0) | 1 ( 0.0) |
| kidney disease | 1 (0.03) | 11 ( 0.4) | 2 ( 0.2) | 13 ( 0.4) |
| antiphospholipid syndrome | 1 (0.03) | 2 ( 0.1) | 0 ( 0.0) | 2 ( 0.1) |
| Preconceptional health behaviors |  |  |  |  |
| Smoking | 0 (0.0) | 540 (21.0) | 182 (16.4) | 771 (20.9) |
| Alcohol consumption | 200 (5.4) | 1451 (61.1) | 709 (63.8) | 2243 (60.9) |
| Daily fruit intake ≥ 2 pieces | 252 (6.8) | 1481 (63.1) | 651 (59.9) | 2275 (61.8) |
| Folic acid (incl. multivitamin) | 0 (0.0) | 1455 (56.5) | 724 (65.2) | 2177 (59.1) |
| Vitamin C (incl. multivitamin) | 0 (0.0) | 568 (22.0) | 301 (27.1) | 868 (23.6) |
| Vitamin D (incl. multivitamin) | 0 (0.0) | 633 (24.6) | 325 (29.3) | 957 (26.0) |
| Calcium (incl. multivitamin) | 0 (0.0) | 532 (20.7) | 282 (25.4) | 813 (22.1) |
| Prenatal health behaviors |  |  |  |  |
| Smoking | 0 (0.0) | 216 ( 8.4) | 66 ( 5.9) | 279 ( 7.6) |
| Alcohol consumption | 200 (5.4) | 24 ( 1.0) | 5 ( 0.5) | 28 ( 0.8) |
| Folic acid (incl. multivitamin) | 0 (0.0) | 2037 (79.1) | 971 (87.4) | 2177 (59.1) |
| Vitamin C (incl. multivitamin) | 0 (0.0) | 1243 (48.3) | 630 (56.7) | 1872 (50.8) |
| Vitamin D (incl. multivitamin) | 0 (0.0) | 1469 (57.0) | 743 (66.9) | 2211 (60.0) |
| Calcium (incl. multivitamin) | 0 (0.0) | 1193 (46.3) | 606 (54.5) | 1798 (48.8) |
| *Obstetrical characteristics* |  |  |  |  |
| Nulliparity | 4 (0.1) | 1137 (44.2) | 504 (45.4) | 1643 (44.6) |
| Recurrent miscarriages ((≥2) | 4 (0.1) | 171 ( 6.6) | 56 ( 5.0) | 227 ( 6.2) |
| Spontaneous conception | 30 (0.8) | 2377 (93.4) | 1028 (92.5) | 3429 (93.1) |
| Time to pregnancy (median (IQR)) (in months) | 529 (14.3) | 3.0 (1.0-6.0) | 3.0 (1.0-6.0) | 3.0 (1.0-6.0) |
| Time to pregnancy (in months) | 529 (14.3) |  |  |  |
| <6 |  | 1551 (72.5) | 753 (74.0) | 2715 (73.7) |
| 6-12 |  | 326 (15.2) | 148 (14.5) | 529 (14.4) |
| >=12 |  | 263 (12.3) | 117 (11.5) | 439 (11.9) |
| Delivery |  |  |  |  |
| Spontaneous | 306 (8.3) | 1729 (75.8) | 820 (74.6) | 2775 (75.3) |
| Induction of labor |  | 417 (18.3) | 208 (18.9) | 684 (18.6) |
| Elective SC |  | 136 ( 6.0) | 71 ( 6.5) | 225 ( 6.1) |
| Gestational age at delivery (days) | 342 (9.3) | 280 (273-285) | 280 (274-286) | 280 (273-285) |
| Gestational age at delivery (weeks) | 312 (8.5) |  |  |  |
| <=24 |  | 9 ( 0.4) | 3 ( 0.3) | 14 ( 0.4) |
| 25-28 |  | 8 ( 0.4) | 5 ( 0.5) | 15 ( 0.4) |
| 29-32 |  | 31 ( 1.4) | 7 ( 0.6) | 44 ( 1.2) |
| 33-36 |  | 60 ( 2.6) | 41 ( 3.7) | 122 ( 3.3) |
| 37-40 |  | 1719 (75.4) | 801 (73.1) | 2730 (74.1) |
| 41-42 |  | 452 (19.8) | 239 (21.8) | 760 (20.6) |
| *Pregnancy complications* |  |  |  |  |
| Spontaneous preterm birth | 306 (8.3) | 66 ( 2.9) | 41 ( 3.7) | 127 ( 3.4) |
| Small for gestational age | 504 (13.7) | 109 ( 5.1) | 53 ( 5.0) | 133 ( 3.6) |
| Gestational diabetes | 264 (7.2) | 118 ( 5.1) | 53 ( 4.8) | 184 ( 5.0) |
| Pregnancy induced hypertension | 264 (7.2) | 143 ( 6.2) | 61 ( 5.5) | 220 ( 6.0) |
| Pre-eclampsia | 264 (7.2) | 54 ( 2.3) | 19 ( 1.7) | 80 ( 2.2) |
| Values are presented as median (IQR) or n (%) | | | | |
| ^1^ Numbers represent characteristics before multiple imputation.  ^2^ Numbers represent characteristics after multiple imputation; due to rounding during the multiple imputation process, numbers do not exactly add up. | | | | |
